# Gradually shifting clinical phenomics in migraine spectrum: a cross-sectional, multicenter study of 5438 patients

**DOI:** 10.1186/s10194-022-01461-5

**Published:** 2022-07-26

**Authors:** Ye Ran, Ziming Yin, Yajun Lian, Yanmei Xu, Yajie Li, Jiale Liu, Qun Gu, Fanhong Yan, Zhaoli Ge, Yu Lian, Dongmei Hu, Sufen Chen, Yangyang Wang, Xiaolin Wang, Rongfei Wang, Xiaoyan Chen, Jing Liu, Mingjie Zhang, Xun Han, Wei Xie, Zhe Yu, Ya Cao, Yingji Li, Ke Li, Zhao Dong, Shengyuan Yu

**Affiliations:** 1 Department of Neurology, International Headache Center, Chinese PLA General Hospital, 100853 Beijing, China; 2 School of Medical Instrument and Food Engineering, University of Shanghai for Science and Technology, 200093 Shanghai, China; 3 Department of Neurology, The First Affiliated Hospital of Zhengzhou University, 450052 Zhengzhou, Henan China; 4 Department of Neurology, Dingyuan General Hospital, 233290 Chuzhou, Anhui China; 5 Diagnostic Ultrasound Centre, The Centre Hospital of Jilin City, 132011 Jilin, China; 6 Department of neurology, The Centre Hospital of Jilin City, 132011 Jilin, China; 7 Department of Neurology, Huzhou First Peolple’s Hospital, 313000 Huzhou, Zhejiang China; 8 Department of Neurology, Linyi jinluo Hospital, 276000 Linyi, Shandong China; 9 Department of Neurology, Shenzhen Second People’s Hospital, 518000 Shenzhen, Guangdong China; 10 Department of Neurology, Inner Mongolia Xing’an League people’s hospital, 137400 Ulanhot, Inner Mongolia China; 11 Department of Neurology, The Second Affiliated Hospital of Shandong First Medical university, 271000 Tian’an, Shandong China; 12 Department of Neurology, Changsha Central Hospital Affiliated to University of South China, 410004 Changsha, Hunan China; 13 Pediatric Center, Chinese PLA General Hospital, 100853 Beijing, China

**Keywords:** Migraine, Migraine aura, Migraine pathophysiology, Factor analysis of mixed data, Decision tree analysis

## Abstract

**Background:**

The aim of the study was to investigate whether MwoA and MwA are different manifestations of a single disease, distinct clinical entities, or located at two poles of a spectrum.

**Methods:**

In this cross-sectional study, 5438 patients from 10 hospitals in China were included: 4651 were diagnosed with migraine without aura (MwoA) and 787 with migraine with aura (MwA). We used a validated standardized electronic survey to collect multidimensional data on headache characteristics and evaluated the similarities and differences between migraine subtypes. To distinguish migraine subtypes, we employed correlational analysis, factor analysis of mixed data (FAMD), and decision tree analysis.

**Results:**

Compared to MwA, MwoA had more severe headaches, predominantly affected females, were more easily produced by external factors, and were more likely to have accompanying symptoms and premonitory neck stiffness. Patients with MwA are heterogeneous, according to correlation analysis; FAMD divided the subjects into three clear clusters. The majority of the differences between MwoA and MwA were likewise seen when typical aura with migraine headache (AWM) and typical aura with non-migraine headache (AWNM) were compared. Furthermore, decision trees analysis revealed that the chaotic MwA data reduced the decision tree’s accuracy in distinguishing MwoA from MwA, which was significantly increased by splitting MwA into AWM and AWNM.

**Conclusions:**

The clinical phenomics of headache phenotype varies gradually from MwoA to AWM and AWNM, and AWM is a mid-state between MwoA and AWNM. We tend to regard migraine as a spectrum disorder, and speculate that different migraine subtypes have different “predominant regions” that generate attacks.

**Supplementary Information:**

The online version contains supplementary material available at 10.1186/s10194-022-01461-5.

## Background

As the first cause of disability in people under 50 years worldwide, migraine has a serious impact on the quality of life for patients and causes a heavy social burden [1]. The International Classification of Headache Disorders, Third Edition (ICHD-III) categorizes migraine into migraine with aura (MwA) and without aura (MwoA) according to the patient’s medical history and clinical manifestations [2]. However, the question of whether MwoA and MwA are different manifestations of a single disease, or distinct clinical entities, or located at two poles of a spectrum, has not yet been completely clarified [3–6].

Although there are many parallels in epidemiology and clinical manifestations between the subtypes, accumulating evidence suggests that the two migraines are distinct in many ways [6–9]. Patients with MwA had thinner cortex bilaterally in the subparietal sulcus, left intraparietal sulcus, and right anterior cingulate, according to structural neuroimaging findings [10]. Resting-state EEGs revealed that MwoA patients had stronger connectivity in the θ band at rest than MwA patients [8]. In addition, depression and anxiety, both closely related to cortical function, were found to be more common comorbidities in MwA than MwoA [11]. The difference in treatment response reflects the differences either. The NMDA receptor antagonist magnesium had a better therapeutic effect in MwA [12]. Lamotrigine, which inhibits cortical spreading depression (CSD), reduced the occurrence of aura substantially [13]. Nevertheless, MwA were less responsive to sumatriptan than MwoA [9]. Moreover, epidemiological studies demonstrated that MwA, but not MwoA, is linked to a higher risk of cardiovascular disease and ischemic stroke [14].

Therefore, this cross-sectional study investigated the demographic and clinical features of large clinical-based cohorts of migraine patients with or without aura from 10 hospitals in China and compared the similarities and differences between migraine subtypes via analysis of multidimensional data, and discussed the gradually shifting characterization in migraine spectrum.

## Methods

### Study design

The study was carried out at headache or neurological clinics of 10 hospitals in China between April 2014 and December 2020. The data were collected using a cross-sectional standardized survey based on ICHD-III [2] or β vision (ICHD-IIIβ) [15] which have been validated in a prospective study [16, 17]. The electronic survey was available online (http://115.28.42.235/Headache) to all qualified researchers [18]. The interview was conducted face-to-face. Two headache specialists revised and confirmed each diagnosis. Chinese PLA General Hospital was responsible for the design and execution of the study.

Patients with headache fulfilling ICHD-III or IIIβ criteria for MwoA, MwA, chronic migraine, or medication-overuse headache derived from migraine were recruited during clinic visits. Patients diagnosed with probable migraine or unable to distinguish between migraine and other headaches were excluded. Informed consent was obtained through an electronic consent from all participants.

 The study protocol complied with the World Medical Association’s Declaration of Helsinki and approved by the Ethics Committee at the Chinese PLA General Hospital and every participating institution, and the ethical number was 2020263.

### Data collection

The standardized electronic survey includes following headache-related data: course, age at onset, type, visual analogue score (VAS), frequency per month, aggravation by activity, duration of attack, family history, main sites, accompanying symptoms, auras, triggers, mitigating factors, and premonitory symptoms (Table 1 & Supplementary Tables). Given that some patients may suffer both MwA and MwoA, our focus in clinical practice was on the “most troublesome headache” according to the patient’s complaint, and recorded clinical features of the type patient suffered mainly.

### Statistical analysis

Statistical analysis was performed using R (3.6.2) and SAS (9.4). Statistical differences of means were calculated using *t-test* or ANOVA, and Tukey HSD was used as post hoc test. χ2 tests and Fisher’s exact test were used for categorical variables, and the Wilcoxon rank sum test was used on numerical data due to nonnormality. Descriptive statistics and logistic regression were carried out. Spearman’s correlation was used for correlational analyses of variables. We performed a factor analysis for mixed data (FAMD) for variables with significant differences of headache phase of all subjects. For the decision tree analysis, all independent variables with significant differences for each comparison were included in the corresponding model.

## Results

### Participation and demography

**Fig. 1:**
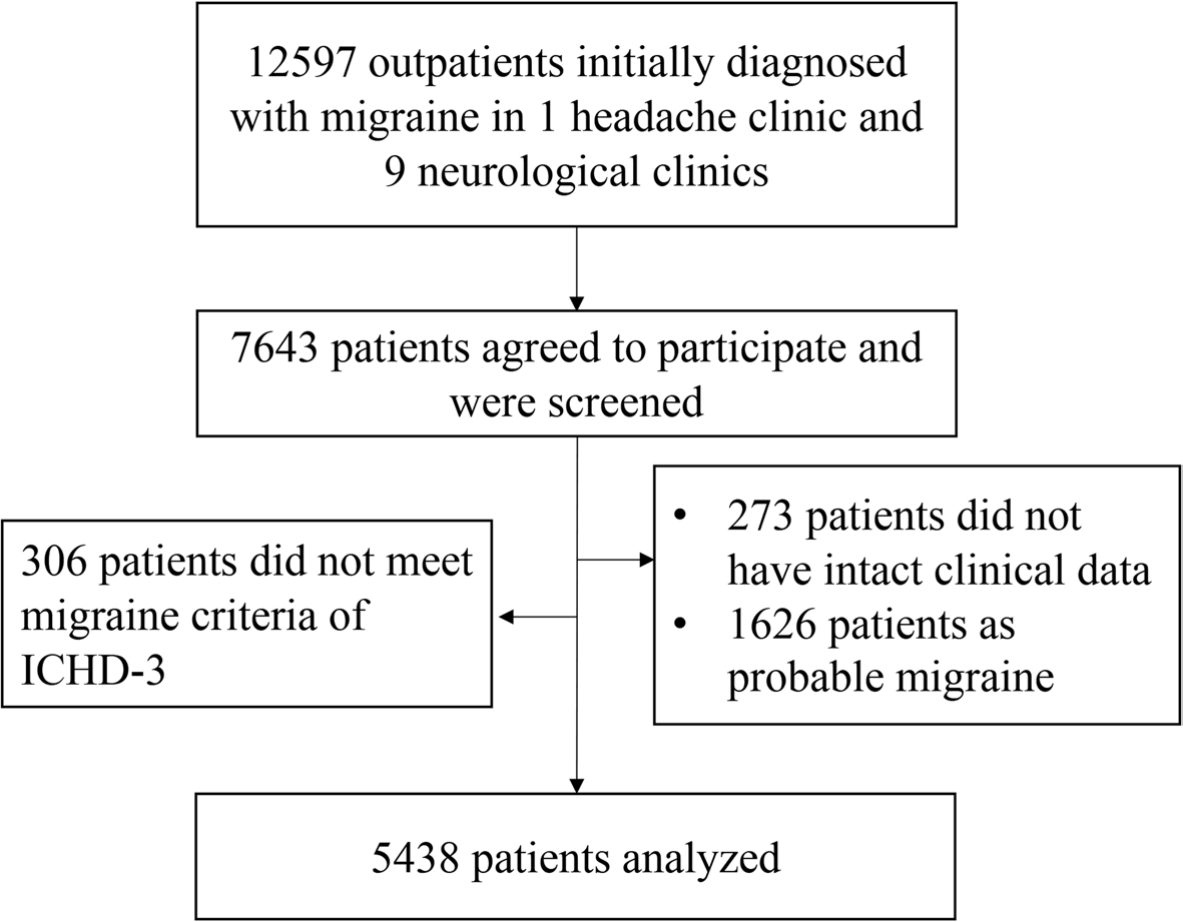
Flow schematic of participant recruitment and data analysis. From April 2014 to December 2020, we consecutively enrolled 12, 597 migraine patients in 10 headache or neurological clinics, of which 7643 agreed to participate in the study. A total of 412 cases were excluded (306 did not meet the diagnostic criteria of migraine, and 273 did not have intact clinical data) in the data verification process, and 1626 cases who were diagnosed as probable migraine. Ultimately, 5438 patients were included for analysis

From April 2014 to December 2020, we consecutively enrolled 12, 597 migraine patients in 10 hospitals, of which 5438 patients were included for analysis (Fig. 1). Of this sample, 4651 (85.53%) patients were diagnosed as MwoA and 787 (14.47%) patients were MwA. Of these, 78.21% were females, with an average of 38.58 years old, 24.69 h of headache duration, and 121.62 months of headache course (Table 1).

**Table 1:** Basic features of demography and Headache

		MwoA	MWA	Total	*p* value
Demographic Features					
Number (%)		4651 (85.53%)	787 (14.47%)	5438	
Gender*	Female (%)	3747 (80.56%)	506 (64.29%)	4253 (78.21%)	< 0.0001
Age*	Mean ± SD (y)	39.33 ± 11.96	34.14 ± 13.67	38.58 ± 12.36	< 0.0001
	≤ 18 (%)	3.83%	12.45%	5.08%	< 0.0001
	19 ~ 40 (%)	50.66%	56.54%	51.51%	
	> 40 (%)	45.52%	31.00%	43.42%	
Age at Onset*	Mean ± SD	28.72 ± 11.94	26.57 ± 13.57	28.40 ± 12.21	< 0.0001
	≤ 18 (%)	22.14%	33.54%	23.83%	< 0.0001
	19 ~ 40 (%)	60.14%	46.85%	58.17%	
	> 40 (%)	15.59%	14.89%	15.49%	
Headache Features					
Migraine-like Headache Type* (%)		91.87%	86.40%	91.08%	< 0.0001
Headache Intensity*	VAS mean ± SD	7.13 ± 1.62	6.68 ± 1.86	7.06 ± 1.66	< 0.0001
	mild (%)	1.18%	5.21%	1.77%	< 0.0001
	moderate (%)	32.79%	39.39%	33.74%	
	severe (%)	66.03%	55.40%	64.49%	
Frequency Per Month*	< 1 d/m (%)	8.54%	27.83%	11.33%	< 0.0001
	1 ~ 15 d/m (%)	70.61%	60.48%	69.14%	
	> 15 d/m (%)	20.86%	11.69%	19.53%	
Aggravation by activity*	none (%)	15.76%	23.51%	16.88%	< 0.0001
	occasionally (%)	8.56%	9.53%	8.70%	
	less than half (%)	7.33%	6.35%	7.19%	
	more than half (%)	68.35%	60.61%	67.23%	
Headache Course*	Mean ± SD (m)	126.85 ± 115.73	90.82 ± 104.57	121.62 ± 114.87	< 0.0001
Headache Duration*	Mean ± SD (h)	25.97 ± 29.83	17.11 ± 37.54	24.69 ± 31.21	< 0.0001
Family History* (%)		46.91%	40.91%	46.05%	0.0003
Main Headache Area					
Tempus* (%)		69.28%	61.50%	68.15%	< 0.0001
Top (%)		39.91%	37.48%	39.55%	0.1989
Forehead (%)		23.35%	23.76%	23.41%	0.8010
Pars orbitalis (%)		12.17%	13.34%	12.34%	0.3551
Face (%)		0.67%	1.14%	0.74%	0.1475
Ear (%)		4.84%	4.19%	4.74%	0.4315
Occiput (%)		35.11%	32.66%	34.76%	0.1810
Neck (%)		2.37%	2.03%	2.32%	0.5669
Accompany Symptoms					
Nausea* (%)		87.49%	78.02%	86.12%	< 0.0001
Vomit (%)		59.49%	57.69%	59.23%	0.3406
Photophobia* (%)		61.97%	55.40%	61.02%	0.0005
Phonophobia* (%)		70.63%	62.77%	69.49%	< 0.0001
Nasal Obstruction* (%)		1.68%	4.07%	2.02%	< 0.0001
Lacrimation* (%)		2.56%	4.32%	2.81%	0.0057
Sweat* (%)		1.16%	5.59%	1.80%	< 0.0001
Palpebral edema* (%)		0.65%	1.02%	0.70%	0.2473
Upset* (%)		6.49%	13.47%	7.50%	< 0.0001

Due to the low occurrence rate, some variables cannot be effectively statistically compared, including Jaw, Blepharoptosis, Myosis

Headache intensity: mild (VSA 1 ~ 3), moderate (VSA 4 ~ 6), severe (VSA 7 ~ 10)

Migraine-like Headache Type include throbbing headache, distending headache, bursting headache

SD: standard deviation, h: hours, d: days, m: months, y: years, d/m: days/month, y: years old

*p value < 0.05

### Comparison of headache characteristics between MwoA and MwA

Compared to MwoA patients, MwA patients had lower headache intensity, shorter duration, lower frequency, and shorter overall course. The proportion of MwoA patients with family history exceeded that of MwA patients. The most common headache site was all in tempus region. MwoA was more often accompanied by nausea, photophobia, and phonophobia than MwA patients. Women accounted for the vast majority of MwoA patients (80.56%), and the ratio of gender in MwA patients was more even (Table 1).

**Fig. 2:**
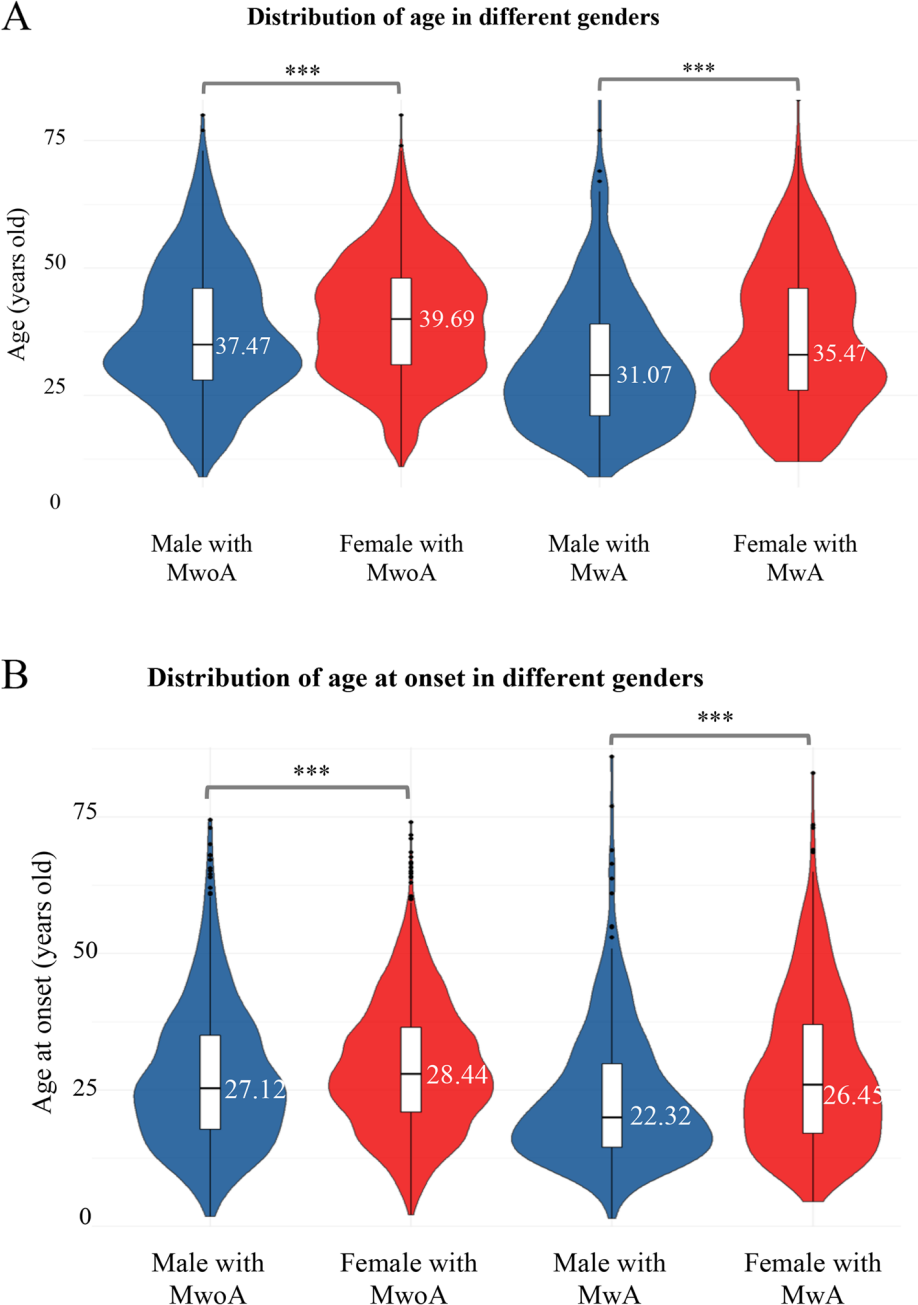
Distribution of age and age at onset in MwoA and MwA patients. Parameters of age and age at onset for different groups are presented as boxplots, which indicate the median and quartiles with whiskers reaching up to 1.5 times the interquartile range. The violin plot shows the kernel probability density, i.e., the width of the shaded area represents the proportion of the data located therein. The average age of MwA group was younger than MwoA group (34.14 ± 13.67 years vs. 39.33 ± 11.96 years, *p* < 0.0001), and the age at onset was also younger in MwA group (26.57 ± 13.57 years vs. 28.72 ± 11.94 years, *p* < 0.0001). *: *p* < 0.05, **: *p* < 0.01, ***: *p* < 0.001

The average of age and age at onset of females were older than those of males in both groups. For the age, females had a more pronounced bimodal distribution of prevalence rates in MwA patients, of approximately 30 and 45 years old, but males had only one peak prevalence rate at approximately 30 years (Fig. 2A). For the age at onset, there was no significant difference between males and females in MwoA patients. The peak incidence rates were all approximately 25 years old, and the distribution of males was just more concentrated. The age at onset of males with MwA was younger, about 20 years old. And among females with MwA, a relatively mild bimodal distribution of prevalence rates of approximately 20 and 40 years was observed (Fig. 2B).

### Triggers and mitigating factors

**Fig. 3:**
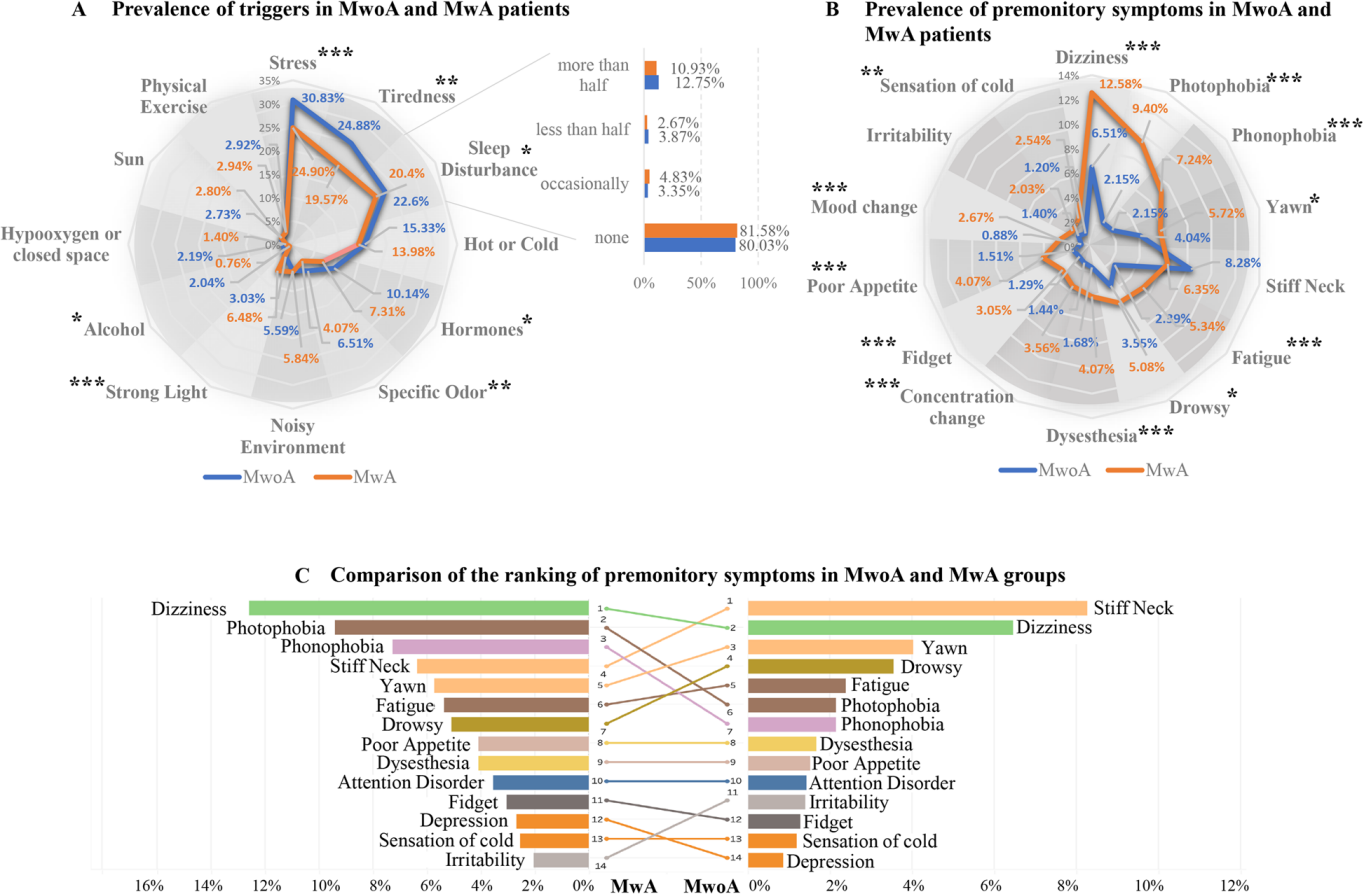
Prevalence of triggers and premonitory symptoms of headache attacks in MwoA and MwA patients. **A** Compared to MwA, MwoA patients were more often associated with most triggers. The only exception was strong light, which played a significantly more common role in MwA. Stress, tiredness, and sleep disturbance were the three most common triggers in both groups. **B** Except for stiff neck and irritability, the incidence of all other premonitory symptoms in MwA patients was higher than MwoA, and most of these triggers exhibited significant differences. **C** Comparison of the ranking of premonitory symptoms in MwoA and MwA groups. Stiff neck, dizzy, and yawn were the most common premonitory symptoms in subjects with MwoA. Dizzy, photophobia, and phonophobia were the premonitory symptoms with the top incidences in MwA. *: *p* < 0.05, ** *p* < 0.01, *** *p* < 0.001

Totally, 69.68% of patients reported at least one trigger. Compared to MwA, MwoA patients more often had triggers (61.50% vs. 71.06%, *p* < 0.0001). Stress, tiredness, and sleep disturbance were the three most common triggers in both types. With the exception of strong light, triggers with significant differences, including stress, tiredness, sleep disturbance, hormones, specific odor and alcohol played a much more common role in MwoA (Fig. 3A; Supplementary Table 1).

There was no significant difference in mitigating factors in each type. The most effective mitigating factors were laying down, dark room, and massage in all patients (Supplementary Table 2).

### Premonitory symptoms (PSs)

Except for stiff neck, the incidence of all PSs in MwA patients was greater than MwoA, and most PSs showed significant differences (Fig. 3B; Supplementary Table 3). Stiff neck (8.28%), dizziness (6.51%), and yawn (4.04%) were the most common PSs in subjects with MwoA, and dizziness (12.58%), photophobia (9.40%), and phonophobia (7.24%) were the PSs with top incidences in MwA (Fig. 3C; Supplementary Table 3).

### Comparison of correlation analysis of headache characteristics between MwoA and MwA

**Fig. 4:**
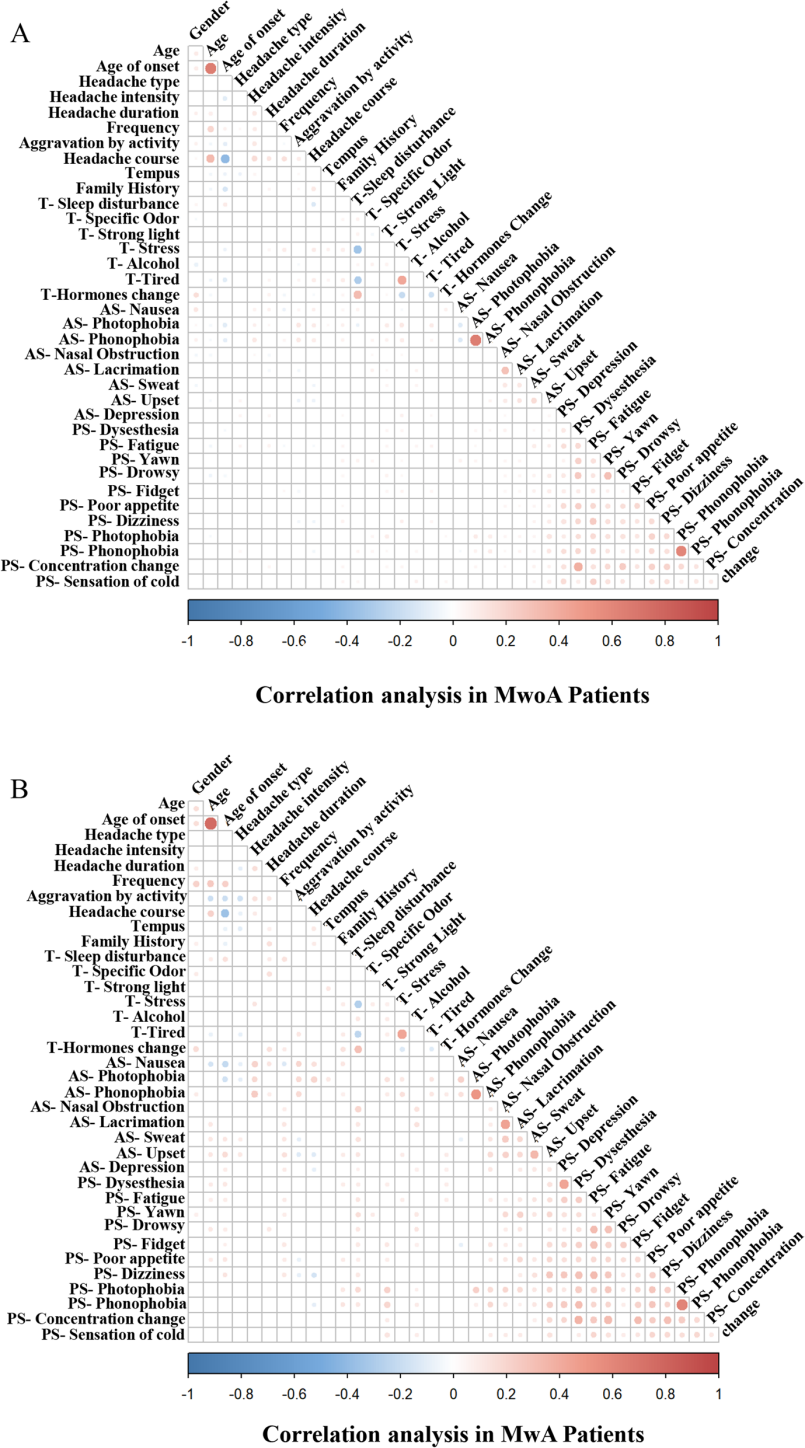
Correlation analyses of headache characteristics in the MwoA and MwA patients. The size of the dot represents the absolute value of the kappa value. Symptoms of nausea and phonophobia were related to greater headache intensity, aggravation by activity, and longer course of headache in MwoA patients, but the correlation coefficient was relatively low (nausea and VAS score, κ = 0.06; nausea and aggravation by activity, κ = 0.04; nausea and course of headache, κ = 0.07; phonophobia and VAS score, κ = 0.11; phonophobia and aggravation by activity, κ = 0.11; *p* < 0.05) (**A**). The positively correlations also existed in MwA patients with relatively high correlation coefficient (nausea and VAS score, κ = 0.2; nausea and aggravation by activity, κ = 0.19; nausea and course of headache, κ = 0.15; phonophobia and VAS score, κ = 0.24; phonophobia and aggravation by activity, κ = 0.17; *p* < 0.05) (**B**). There are less correlations between triggers such as sleep disturbance, specific odor, hormones, with longer duration or higher frequency of headaches in MwoA patients (**A**). However, the positively correlation coefficients in MwA patients were relatively high (sleep disturbance, κ = 0.10; specific odor, κ = 0.14; hormones, κ = 0.14; *p* < 0.05) (**B**). And migraine-like headaches in MwA patients were positively correlated with longer duration of headache attack and aggravation by activity (duration, κ = 0.10; aggravation by activity, κ = 0.16) (**B**). Kappa values with no significant differences are not shown. T: trigger; AS: accompanying symptom; PS: premonitory symptom; MF: mitigating factor

The occurrence of symptomatic nausea and phonophobia in MwA patients positively correlated with greater headache intensity, aggravation by activity, and longer course of headache (Fig. 4B). The positively correlations also existed in MwoA patients, but the correlation coefficient was relatively low (Fig. 4A). Triggers such as sleep disturbance, specific odor, and hormones in MwA patients were positively correlated with longer duration of headache attack (Fig. 4B). However, these correlations in MwoA patients were less (Fig. 4A). In addition, Migraine-like headaches in MwA patients were positively correlated with longer duration of headache attack and aggravation by activity (Fig. 4B).

### Factor analysis for mixed data (FAMD)

**Fig. 5:**
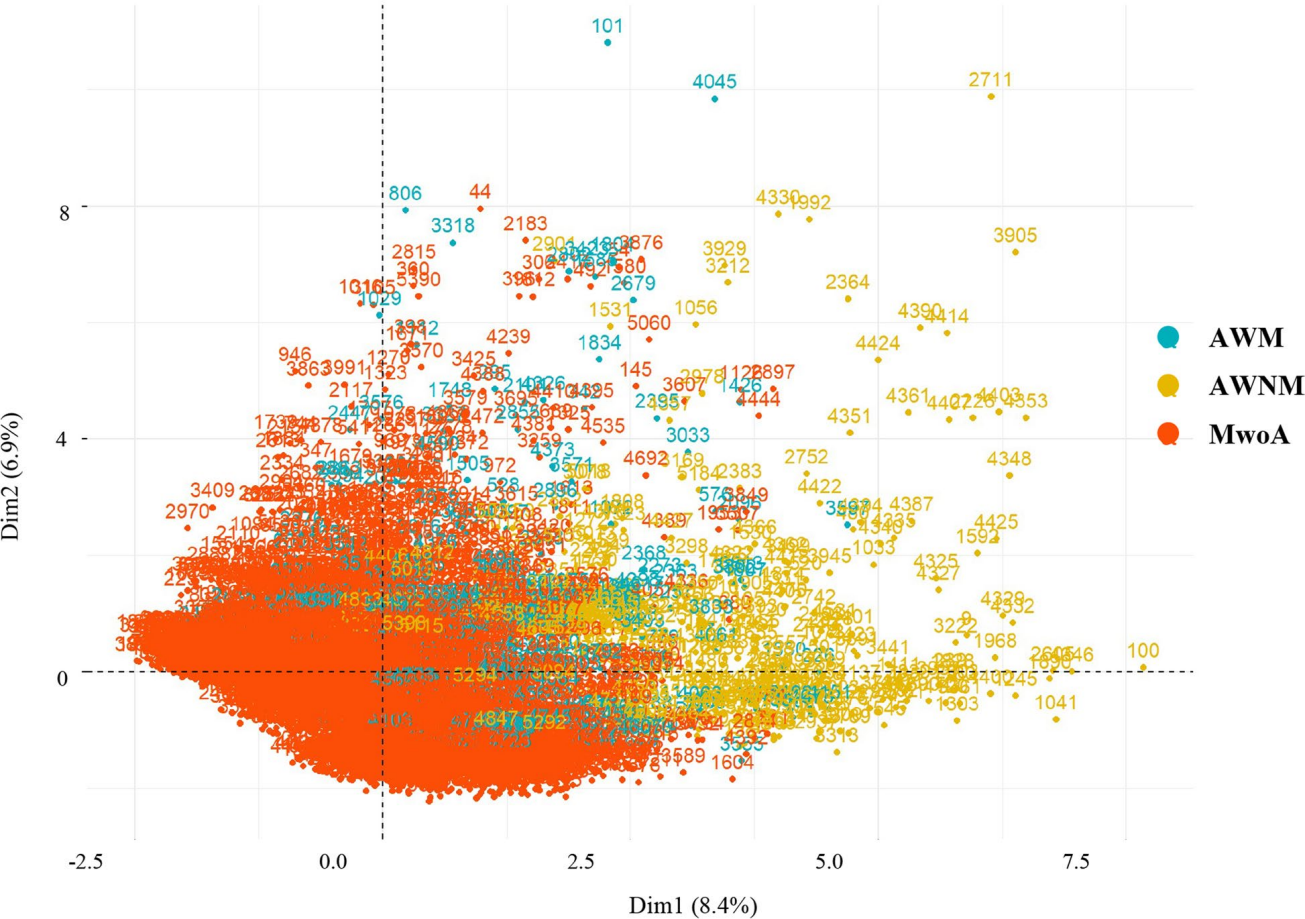
Graphical results of the factor analysis for mixed data (FAMD). The x- and y-axes represent the 2 first dimensions of the FAMD (these 2 dimensions explained 15.3% of the point variability). Subjects were subdivided into three separate clusters (red, green, and yellow dots), which represented three subtypes of migraine (MwoA, AWM, and AWNM)

According to the ICHD-II [19], MwA can be further classified into typical aura with migraine headache (AWM) and typical aura with non-migraine headache (AWNM). We incorporated characteristic indicators related to headache period into the FAMD analysis, including headache type, VAS score, frequency, aggravation by activity, course of headache, duration of headache, main headache area, accompanying symptoms, family history, age at onset, and gender, and further labeled MwA as AWM or AWNM. Subjects were subdivided into three separated clusters, which represented three subtypes of migraine (MwoA, AWM, and AWNM) (Fig. 5).

### Comparison of headache related characteristics in AWNM, AWM, and MwoA

**Fig. 6:**
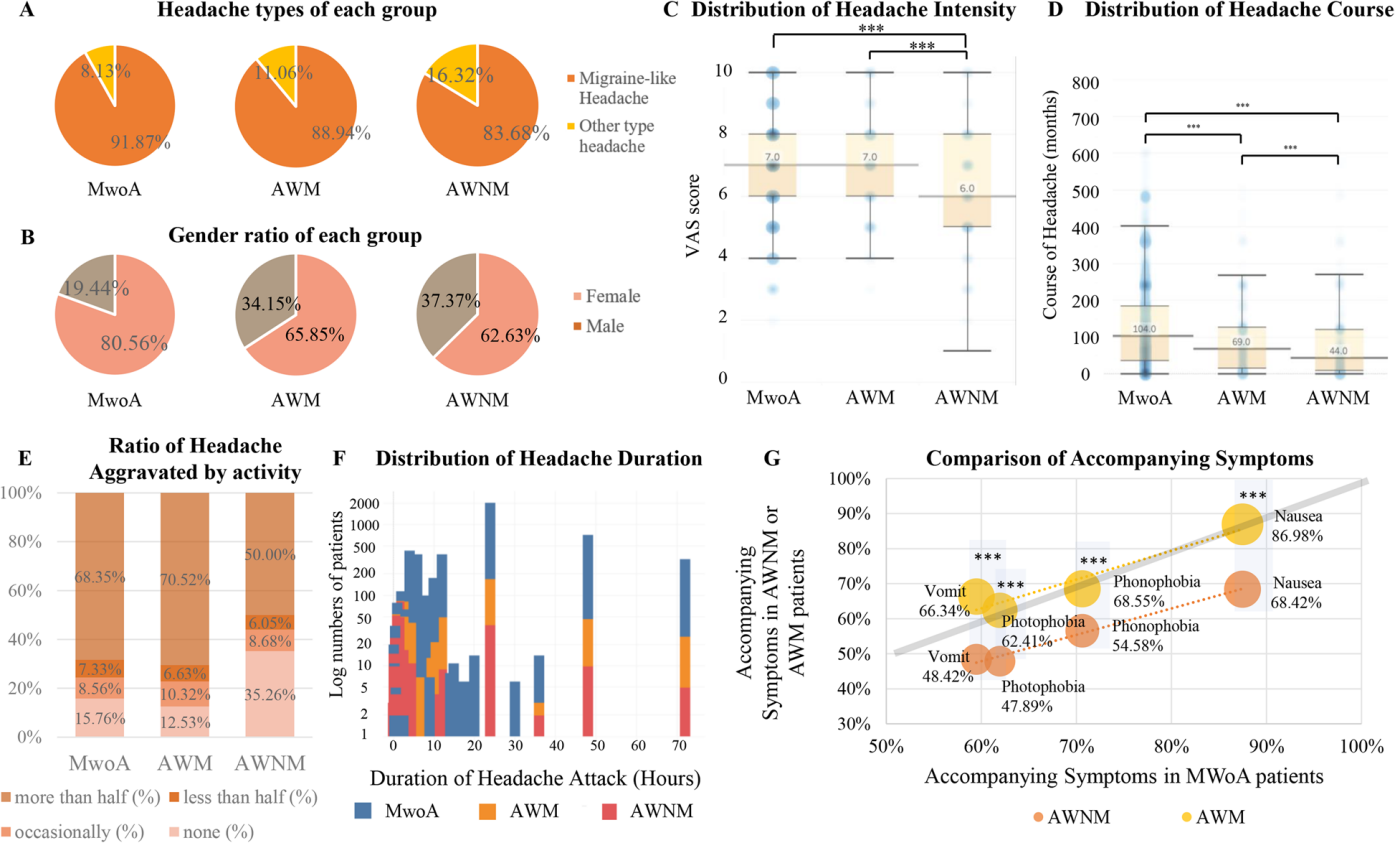
Comparison of related characteristics of AWNM, AWM, and MwoA. Migraine-like headache occupied absolute majorities across all groups, and AWNM patients contributed to a much larger percentage of other headache types (16.32%) (**A**). The proportion of females was higher in MwoA than AWM and AWNM (**B**). MwoA and AWM patients had more severe headaches (**C**), longer headache course (**D**) than AWNM patients. The medians and quartiles are presented as boxplots with whiskers with a maximum 1.5 interquartile range (IQR). The circles illustrate kernel probability density, and the color depth of the circle represents the proportion of the data located therein (**C** and **D**). Compared to AWNM, headache attacks in MwoA and AWM patients were more likely to be aggravated by activity (**E**). The Duration of headache attacks was significantly different across all types. MwoA patients had the longest duration of headache, followed by AWM patients, and the shortest was AWNM patients (25.97 ± 29.83 h in MwoA, 21.75 ± 27.34 h in AWM, 12.14 ± 45.53 h in AWNM). The Y-axis represents the logarithm of patient numbers with corresponding headache duration (**F**). The incidence of all accompanying symptoms in AWM patients was higher than AWNM patients with significant differences, and the incidences in AWM patients were similar to MwoA patients. The size of the circle represents the incidence of the accompanying symptoms in AWNM or AWM patients. The closer the circle is to the gray diagonal, the closer the incidence of accompanying symptoms is to MwoA patients (**G**). * *p* < 0.05, ** *p* < 0.01, *** *p* < 0.001

Migraine-like headache occupied an absolute majority across all types, but AWNM patients contributed to a much larger percentage of other types of headaches (Fig. 6A). The gender ratio of the AWNM and AWM patients were similar (Fig. 6B). AWM patients had more severe headaches, longer duration of headache attacks, and slightly longer headache course than AWNM patients (Fig. 6C, D and F). Compared to AWNM, activity was more likely to aggravated headache attacks in MwoA and AWM patients (Fig. 6E). The incidence of all accompanying symptoms in patients with AWM was higher than that of AWNM, with significant differences (Fig. 6G). The age and age at onset of AWNM patients were slightly higher than AWM patients. And the incidence of almost all kinds of triggers in patients with AWM were higher than in AWNM patients, with significant differences in specific odor. The incidence of neck stiffness, as a premonitory symptom, was higher in patients with AWM, and poor appetite was more common in AWNM. Other premonitory symptoms were similar between the subtypes of MwA (Supplementary Table 4). The results of comparisons between AWNM and MwoA were shown in Supplementary Table 5.

### Differences in multivariate logistic analysis

**Fig. 7:**
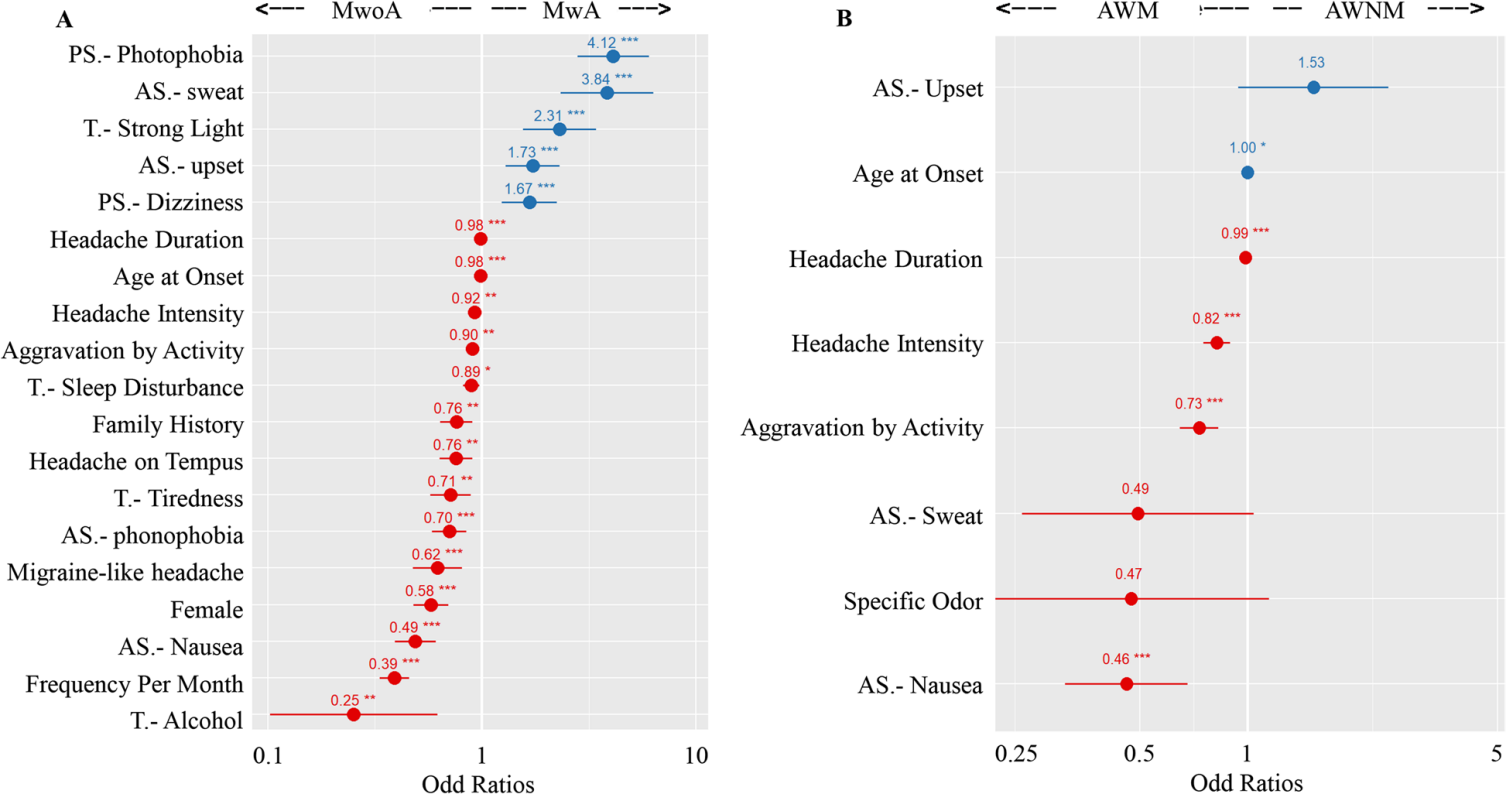
Forest plot of characteristics of migraine that distinguish MwoA and MwA (**A**), AWM and AWNM (**B**). Variable-specific odds ratios (95% confidence intervals) are denoted by dots (lines). The detailed results of the binary logistical regression model can be found in Supplementary Tables 6 and 7. *P-value < 0.05, **P-value < 0.01, ***P-value < 0.001

The headache duration, frequency, intensity, migraine-like attacks, aggravated by activity, gender ratio, age at onset, family history, trigger factors (including sleep disturbance, tiredness, alcohol, strong light), accompany symptoms (including nausea, phonophobia, sweat, upset), and premonitory symptoms (including photophobia, dizziness), remained significant in the multivariate model in comparison between MwoA and MwA (Fig. 7 A; Supplementary Table 6). Similarly, headache duration, intensity, aggravated by activity, trigger factors (including specific odor), accompany symptoms (including sweat, nausea, and upset), remained significant in the multivariate model in comparison between AWM and AWNM (Fig. 7B; Supplementary Table 7).

### Decision tree analysis

**Fig. 8:**
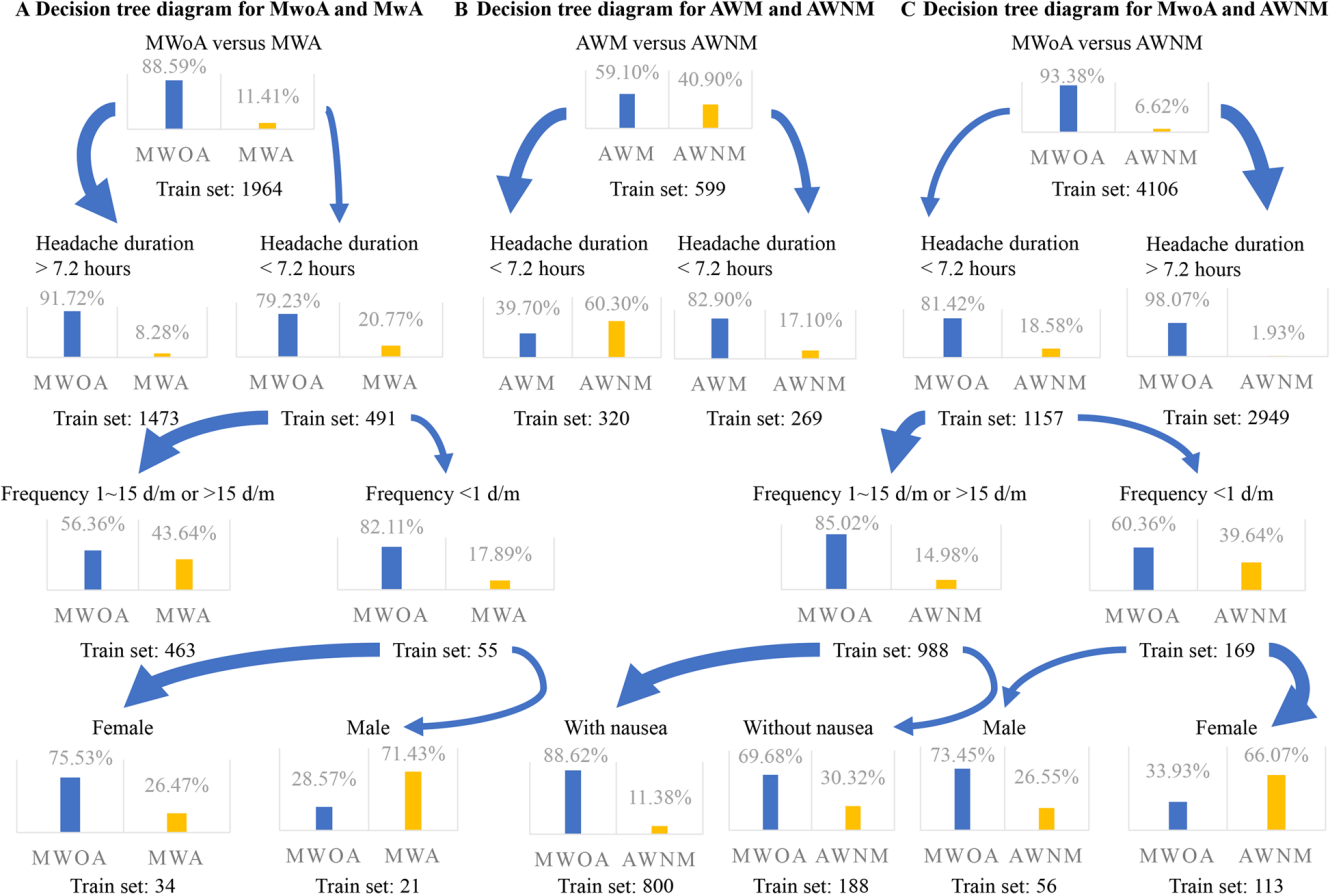
Decision tree diagram for the distinguishing of migraine subtypes. In individuals with a headache lasting shorter than 7.2 h, the proportion of MwA in the training set increased to 20.77% from 11.41%. Among them, patients who experienced headaches more than once a month had a 43.64% chance of developing MwA (**A**). The only variable that ultimately distinguished AWM and AWNM was the duration of headache. In patients with a headache duration shorter than 7.2 h, the proportion of AWNM patients in the training set grew from 40.90–60.30%, while AWNM patients accounted for only 17.10% in patients with a headache duration longer than 7.2 h (**B**). For distinguishing MwoA from AWNM, the variables were duration of headache, frequency, and whether it was accompanied by nausea. The proportion of AWNM patients in the training set boosted from 6.62–18.58% in patients with headaches lasting less than 7.2 h, while AWNM patients accounted for only 1.93% in patients with headaches lasting more than 7.2 h. If the frequency of headaches was less than 1 day/month, the proportion of AWNM patients increased to 39.64%. There was an 88.62% probability of MwoA in patients with headache duration less than 7.2 h, headache frequency greater than 1 day/month, and accompanied with nausea (**C**). d/m: days/month

To classify migraine subtypes and identify critical variables, we employed decision tree classification. In the decision tree analysis, all variables having a significant difference in the pairwise comparison between each subtype were implicated. The noteworthy differences between MwoA and MwA were headache duration, headache frequency, and gender. The only variable that ultimately distinguished AWM and AWNM was the duration of headache. The critical variables in separating MwoA from AWNM were headache duration, frequency, and whether or not it was accompanied with nausea symptoms (Fig. 8). These findings indicate that the characteristics listed above play a larger role in determining migraine subtype.

The decision tree’s Youden’s index and area under the curve (AUC) for distinguishing MwoA and MwA was very low, indicating that the decision tree model had a poor capacity to identify MwoA and MwA. When MwA was split into AWNM and AWM and compared, the Youden’s index that classified the AWNM and AWM increased to 0.4593, which increased to 0.3179 in that classified MwoA and AWNM, and the AUC improved to 0.7297 and 0.8137, separately (Supplementary Table 8).

## Discussion

To the best of our knowledge, this is the first large-scale cross-sectional study of migraine headache characteristics from multiple centers, allowing us to further refine the clinical correlations between MwA and MwoA. Based on the disparities in headache characteristics, accompanying symptoms, triggers, and premonitory symptoms between the subtypes, we propose that MwoA and MwA are not distinct entities, but rather have tightly coupled relationships. Correlation analysis revealed that MwA patients were heterogeneous, and FAMD separated the subjects into three distinct clusters. Decision trees analysis revealed that the chaotic MwA data reduced the decision tree’s accuracy in distinguishing MwoA from MwA. All the results suggest that MwoA and AWNM being at opposite ends of a spectrum, while AWM is most likely in the middle.

### Comparison of MwoA and MwA

#### Proportion of MwoA to MwA

The ratio of MwoA to MwA was 5.9:1 in our study, which is quite consistent with the results of our previous study [20]. The ratio is also similar to the Taipei Area (7.0:1) [21] and Japan (7.4:1) [22], but different from Europe (1.6:1 ~ 3.8:1) [23–26]. This variation could be due to a genetic predisposition.

#### Differences in gender and hormones

Our subjects revealed a significant female preponderance. Another study in a single center in southwestern China found a similar gender distribution from outpatient population [27]. Previous population-based research in Asia and Europe have also revealed that the MwoA group has a high female/male ratio (4.9:1 ~ 2.2:1) [4, 21, 22, 25]. Notably, MwA revealed a closer sex ratio compared to MwoA.

The distribution of age and age at onset showed an excess of female patients with MwoA in reproductive age, and female patients with MwA were more common in younger and older populations, which is consistent with previous reports [4, 27]. Ovarian steroid hormones are also an important trigger in migraine. We observed a clear difference in the percent of headache attacks induced by menstruation or pregnancy between MwoA and MwA patients, which is consistent with prior data [28, 29]. MwoA seemed to alleviate more during pregnancy than MwA, according to Granella et al. [29]. All of these findings suggest that estrogen fluctuation is more important in MwoA than MwA itself. According to previous researches, estrogen affects neuroexcitability and regulates the occurrence of migraine by affecting neurotransmitters such as 5-hydroxytryptamine (5-HT) [30], γ-aminobutyric acid (GABA) [31], calcitonin-gene related peptide (CGRP) [32], opioids [33], and norepinephrine [34], and these neurotransmitters are primarily distributed in the raphe nuclei pontis, periaqueductal gray (PAG), ventral tegmental area (VTA), hypothalamus, and the ventromedial side of the thalamus, which suggests that MwoA is more closely related to the hypothalamus, thalamus, and brainstem than MwA.

#### Differences in triggers

MwoA patients were more likely to be connected with triggers than MwA patients, which was consistently reported [35, 36]. Some researchers found that patients with both MwoA and MwA had more triggers for MwoA attacks than WMA attacks [37]. According to previous findings, sleep-wake cycle disturbance [38], changes in mood [39], stress [40], and fears to specific odor [41] are closely related to hypothalamic function, limbic structures and descending modulation of the brainstem. The difference of triggers suggested different activity levels of these structures between the two subtypes. Strong light was the only trigger with a higher incidence in MwA patients in the current study, which is consistent with previous research [4]. This phenomenon may be due to the hyperresponsiveness of visual cortex in MwA [6]. However, some researchers argued that flickering lights were more likely to be a premonitory symptom associated with abnormal brain activity rather than a trigger [42]. Therefore, the high incidence of strong light as a trigger in patients with MwA may correlate with the high incidence of premonitory photophobia.

#### Differences in premonitory symptoms

The PSs are the beginning of alternations in brain function prior to headache attack [42], which suggests that some brain areas are initiatively activated. Based on our results, MwA patients were more likely to be accompanied by PSs, and the most common PSs in MwA and MwoA were different. Neck stiffness, as the most common symptom of MwoA, is associated with activation of the periaqueductal gray, trigeminocervical complex, hypothalamus, and thalamus [43, 44]. Photophobia and phonophobia are more common in MwA and are more likely to be associated with cortical dysfunction. Therefore, the differences in PSs suggest that brain areas involved in MwoA and MwA are not exactly the same, which may provide important insight into the mechanisms of migraine.

#### From MwoA to MwA, change happens gradually

Previous researches on migraine chronicity considered migraine as a spectrum disorder, in which the clinical and pathophysiological features of migraine may progress over time [45, 46]. Based on the multidimensional clinical information of distinct migraine subtypes, the current investigation looked into whether migraine was a spectrum condition. According to our results, accompanying symptoms, triggers, and migraine-like headaches were more common in MwoA. Patients with MwoA generally suffered a more severe headache, a higher frequency, and a longer course than MwA. The correlation analysis found that headache characteristics, including accompanying symptoms, triggers, and migraine-like headache, positively correlated with the intensity, frequency, or course of headache in MwA patients. However, these positive correlations did not occur among MwoA patients. Therefore, we speculated that patients with MwA were heterogeneous.

FAMD put participants into three groups, each representing one of three migraine subtypes (MwoA, AWM, and AWNM). The green dots presented AWM patients who were positioned between the yellow AWNM and the red MwoA, and the AWNM and MwoA boundaries were relatively apparent (Fig. 5). These findings suggest that AWM is a compromise between AWNM and MwoA. The differences in basic headache aspects between MwoA and MwA were also noticed when AWM and AWNM were compared. Patients with AWM were more similar to those with MwoA, and the differences between MwoA and AWNM were clearer. And the chaotic MwA data affected the decision tree’s accuracy in identifying MwoA from MwA, according to decision tree analysis. When MwA was separated into AWM and AWNM and compared, the Youden’s index of the decision trees grew dramatically.

These findings suggest that MwA patients should be separated into AWM and AWNM groups, with AWM as a transitional condition. Changes in headache features develop gradually from MwoA to AWM and AWNM. More research is needed to investigate whether patients with MwA should be reclassified in the further diagnostic criteria based on clinical manifestations with vs. without migraine-like headache, as defined in the ICHD-II [19]. Understanding these subtypes could be crucial in guiding future migraine research and improving therapeutic outcomes.

#### Implications for pathophysiology of MwA and MwoA

The pathophysiology of aura is commonly acknowledged CSD [47], which is a spontaneous activation of the brain that propagates slowly and continuously. However, the possibility that silent CSD is the underlying etiology of MwoA is much less certain [48]. The hypothalamus, a critical integrator between external stimuli and internal metabolic and endocrinological variables, has been suggested to play a pivotal role in the genesis of headache attacks in MwoA [49, 50]. Internal and external factors may lead to alterations in hypothalamic activity, which may influence pain processing pathways and facilitate trigeminal pain perception, resulting in a headache attack [50–52].

Our findings showed that MwoA patients had more severe headaches, a stronger link to estrogen, were more easily produced by external causes, and were more likely to have accompanying symptoms and premonitory symptoms. As previously stated, all of these differences suggest that MwoA has a closer relationship to subcortical brain dysfunction, such as the hypothalamus, thalamus, and descending modulation system of the brainstem, than MwA, especially AWNM, and that MwA is more closely aligned with cortical dysfunction. We hypothesize that in MwA and MwoA, the activation degree of different brain regions or neural networks may be different. Different subtypes may have different “predominant regions” in the brain that generate migraine attacks. Notably, the existence of an intermediate type of AWM between MwoA and AWNM suggests that the two generators are not entirely separated, but that a progressive process linkage may exist. Therefore, we tend to consider migraine as a spectrum disorder, with MwoA and AWNM as the two poles.

#### Strengths and Limitations

One strength of this multicenter study is the large number of subjects with multidimensional data on headache characteristics. To our knowledge, this study is the first application of factor analysis of mixed data and decision tree to patients with migraine to distinguish subtypes and investigate the dominant features. Although the probable migraine probably lies on the spectrum of migraine, we excluded the subtype in our study to obtain a more obvious consequence. There are also some limitations in our research. This research was based on outpatient data, not a population-based survey. While patients with more severe symptoms or higher educational attainment are more likely to seek hospital care for their headache and agree to participate the survey. And the study collected the headache data in outpatient clinics without the use of headache diaries, which cannot avoid recall bias. Some patients with both MwA and MwoA, may confound the characters of headache attacks. As we known, a very small percentage of patients have more than one form of headache, per prior studies. Only 9% of Danish migraine patients had both MwA and MwoA, compared to a total of 36% who had MwA only [53]. In comparison to the population of Europe, East Asians have a much lower prevalence of MwA. The ratio of MwoA to MwA was 5.9 ~ 7.4:1 in East Asian population [20–22]. Therefore, the proportion of comorbidities with both MwA and MwoA in our study should be smaller than that in European population, further minimizing its influence on the study’s findings. In addition, this study was a cross-sectional study that could not track changes in clinical manifestations of migraine patients.

## Conclusions

Compared to MwA, MwoA had more severe headaches, predominantly affected females, was more easily induced by external factors, and was more likely to have accompanying symptoms and premonitory neck stiffness. Correlation analysis suggested that patients with MwA were heterogeneous. FAMD grouped the subjects into three clusters. Most differences between MwoA and MwA were also identified in the comparison between AWM and AWNM. The chaotic MwA data affected the decision tree’s accuracy in differentiating MwoA from MwA, which was significantly increased by splitting MwA into AWM and AWNM, according to decision trees analysis. Changes in headache characteristics develop progressively from MwoA to AWM and AWNM, and AWM is a mid-state between MwoA and AWNM. Furthermore, the differences in subtypes features suggest that MwoA is more directly associated with hypothalamic and thalamic functions, as well as descending modulation of the brainstem, than MwA, especially AWNM, and that MwA is strongly associated with cortical dysfunction. We tend to think of migraine as a spectrum disorder, with different migraine subtypes having different “predominant regions” that generate episodes.

## Supplementary Information

**Additional file 1: Table 1. **Prevalence of triggers of headache in MWoA and MWA. **Table 2.** Prevalence of mitigating factors of headache in MWoA and MWA. **Table 3. **Prevalence of premonitory symptoms of headache in MWoA and MWA. **Table 4. **Comparison between AWNM and AWM. **Table 5. **Comparison between MWoA and AWNM. **Table 6.** Multivariate analysis of clinical characteristics for distinction of MwoA and MwA. **Table 7.** Multivariate analysis of clinical characteristics for distinction of AWM and AWNM. **Table 8.** Fit statistics for the selected tree.

## Data Availability

The datasets used and/or analyzed during the current study are available from the corresponding authors on reasonable request and with permission of Chinese PLA General Hospital.

## Abbreviations

MwoA: Migraine without aura
MwA: Migraine with aura
AWM: Typical aura with migraine headache
AWNM: Typical aura with non-migraine headache
FAMD: Factor analysis of mixed data
CSD: Cortical spreading depression
ICHD: International classification of headache disorders
VAS: Visual analogue score
PSs: Premonitory symptoms
AUC: Area under curve
GABA: γ-aminobutyric acid
CGRP: Calcitonin-gene
RP: Related peptide
5-HT: Hydroxytryptamine
PAG: Periaqueductal gray
VTA: Ventral tegmental are
