# Supplementary material for: Gradually shifting clinical phenomics in migraine spectrum: a cross-sectional, multicenter study of 5438 patients
Source: J Headache Pain. 2022 Jul 26;23(1):89. doi: 10.1186/s10194-022-01461-5 (PMC9327365; doi:10.1186/s10194-022-01461-5)
Supplement: Supplementary file 1 — Additional file 1: Table 1. Prevalence of triggers of headache in MWoA and MWA. Table 2. Prevalence of mitigating factors of headache in MWoA and MWA. Table 3. Prevalence of premonitory symptoms of headache in MWoA and MWA. Table 4. Comparison between AWNM and AWM. Table 5. Comparison between MWoA and AWNM. Table 6. Multivariate analysis of clinical characteristics for distinction of MwoA and MwA. Table 7. Multivariate analysis of clinical characteristics for distinction of AWM and AWNM. Table 8. Fit statistics for the selected tree. [file 10194_2022_1461_MOESM1_ESM.docx]

**Supplementary Table 1. Prevalence of triggers of headache in MWoA and MWA.**

| Trigger |  | MWoA | MWA | Total | *p* value |
| --- | --- | --- | --- | --- | --- |
| Stress* (%) |  | 30.83% | 24.90% | 29.97% | 0.0008 |
| Tiredness* (%) | | 24.88% | 19.57% | 24.11% | 0.0013 |
| Sleep Disturbance* | none (%) | 80.03% | 81.58% | 80.25% | 0.0332 |
|  | occasionally (%) | 3.35% | 4.83% | 3.57% |  |
|  | less than half (%) | 3.87% | 2.67% | 3.70% |  |
|  | more than half (%) | 12.75% | 10.93% | 12.49% |  |
| Hot or Cold (%) | | 15.33% | 13.98% | 15.13% | 0.3274 |
| Hypooxygen or closed space (%) | | 2.19% | 1.40% | 2.08% | 0.1480 |
| Sun (%) | | 2.73% | 2.80% | 2.74% | 0.9719 |
| Hormones* (%) | | 10.14% | 7.31% | 9.80% | 0.0466 |
| Specific Odor* (%) | | 6.51% | 4.07% | 6.16% | 0.0082 |
| Noisy Environment (%) | | 5.59% | 5.84% | 5.63% | 0.7742 |
| Strong Light* (%) | | 3.03% | 6.48% | 3.53% | < 0.0001 |
| Alcohol* (%) |  | 2.04% | 0.76% | 1.86% | 0.0139 |
| Physical Exercise (%) | | 2.92% | 2.94% | 2.92% | 0.8854 |
| Trigger* (%) | | 71.06% | 61.50% | 69.68% | < 0.0001 |
| Due to the low occurrence rate, some variables cannot be effectively statistically compared, including coffee, cheese, monosodium glutamate, nitro, cough, sexual activity, menophause, position, starve, catch a cold.  Hormones induced headache included migraine induced by menstruation or pregnancy. The incidence of trigger hormones was calculated in female subjects.  MWoA : migraine without aura; MWA : migraine with aura.  * *p* value < 0.05. | | | | | |

**Supplementary Table 2. Prevalence of mitigating factors of headache in MWoA and MWA.**

| Mitigating Factor |  | MWoA | MWA | Total | *p* value |
| --- | --- | --- | --- | --- | --- |
| Laying down (%) |  | 66.31% | 66.96% | 66.40% | 0.7191 |
| Dark room (%) |  | 43.13% | 44.22% | 43.29% | 0.5689 |
| Massage (%) |  | 25.65% | 23.13% | 25.29% | 0.1318 |
| Hot Compress (%) |  | 5.01% | 3.68% | 4.82% | 0.1085 |
| Cold Compress (%) |  | 2.45% | 3.05% | 2.54% | 0.3235 |
| Fast Walk (%) |  | 1.25% | 0.64% | 1.16% | 0.1380 |
| Exercise (%) |  | 1.31% | 0.89% | 1.25% | 0.3244 |
| Pregnant (%) |  | 2.30% | 1.38% | 2.19% | 0.1881 |
| Due to the low occurrence rate, some variables cannot be effectively statistically compared, including stand.  The incidence of mitigating factor pregnant was calculated in female subjects.  MWoA : migraine without aura; MWA : migraine with aura.  * *p* value < 0.05. | | | | | |

**Supplementary Table 3. Prevalence of premonitory symptoms of headache in MWoA and MWA.**

| Premonitory Symptom | | MWoA | MWA | Total | *p* value |  |
| --- | --- | --- | --- | --- | --- | --- |
| Dizziness* (%) |  | 6.51% | 12.58% | 7.39% | < 0.0001 |  |
| Photophobia* (%) |  | 2.15% | 9.40% | 3.20% | < 0.0001 |  |
| Phonophobia* (%) |  | 2.15% | 7.24% | 2.89% | < 0.0001 |  |
| Yawn* (%) |  | 4.04% | 5.72% | 4.28% | 0.0318 |  |
| Stiff Neck (%) |  | 8.28% | 6.35% | 8.00% | 0.0657 |  |
| Fatigue* (%) |  | 2.39% | 5.34% | 2.81% | < 0.0001 |  |
| Drowsy* (%) |  | 3.55% | 5.08% | 3.77% | 0.0365 |  |
| Dysesthesia* (%) |  | 1.68% | 4.07% | 2.02% | < 0.0001 |  |
| Concentration change* (%) | | 1.44% | 3.56% | 1.75% | < 0.0001 |  |
| Fidget* (%) |  | 1.29% | 3.05% | 1.54% | 0.0002 |  |
| Poor Appetite* (%) |  | 1.51% | 4.07% | 1.88% | < 0.0001 |  |
| Mood change* (%) |  | 0.88% | 2.67% | 1.14% | < 0.0001 |  |
| Irritability (%) |  | 1.40% | 2.03% | 1.49% | 0.1735 |  |
| Sensation of cold* (%) | | 1.20% | 2.54% | 1.40% | 0.0031 |  |
| Due to the low occurrence rate, some variables cannot be effectively statistically compared, including loquacity, thirsty, constipation, diarrhea, osmophobia, diuresis, dysphasia, craving food, over activity, edema.  MWoA : migraine without aura; MWA : migraine with aura.  * *p* value < 0.05. | | | | | | |

**Supplementary Table 4. Comparison between AWNM and AWM**

|  |  | AWNM | AWM | *p* value |
| --- | --- | --- | --- | --- |
| Demographic Features | | | | |
| Number (%) |  | 380 (48.28%) | 407 (51.72%) |  |
| Age* | Mean ± SD (y) | 35.46±14.65 | 32.92±12.59 | 0.0092 |
|  | ≤ 18 y (%) | 12.63% | 12.29% | 0.0007 |
|  | 19~40 y (%) | 52.37% | 60.44% |  |
|  | > 40 y (%) | 35.00% | 27.27% |  |
| Gender | Female (%) | 62.63% | 65.85% | 0.3777 |
| Age at Onset* | Mean ± SD (y) | 28.31±14.92 | 24.94±11.97 | 0.0005 |
|  | ≤ 18 y (%) | 33.16% | 37.10% | 0.0027 |
|  | 19~40 y (%) | 46.32% | 51.84% |  |
|  | > 40 y (%) | 20.53% | 11.06% |  |
| Headache Features | | | | |
| Migraine-like Headache Type* (%) | | 83.68% | 88.94% | 0.0315 |
| Headache Intensity* | VAS mean ± sd | 6.24±1.97 | 7.09±1.65 | < 0.0001 |
|  | mild (%) | 9.47% | 1.23% | < 0.0001 |
|  | moderate (%) | 44.74% | 34.40% |  |
|  | severe (%) | 45.79% | 64.37% |  |
| Frequency Per Month | < 1 d/m (%) | 30.53% | 25.31% | 0.1293 |
|  | 1~15 d/m (%) | 56.84% | 63.88% |  |
|  | > 15 d/m (%) | 12.63% | 10.81% |  |
| Aggravation by activity* | none (%) | 35.26% | 12.53% | < 0.0001 |
|  | occasionally (%) | 8.68% | 10.32% |  |
|  | less than half (%) | 6.05% | 6.63% |  |
|  | more than half (%) | 50.00% | 70.52% |  |
| Headache Course* | Median (m) | 45.00 | 72.00 | 0.0061 |
| Headache Duration* | Mean ± SD (h) | 12.14±45.53 | 21.75±27.34 | 0.0003 |
| Family History (%) | | 36.05% | 45.45% | 0.1417 |
| Main Headache Area | | | | |
| Tempus (%) |  | 58.95% | 63.88% | 0.1551 |
| Top (%) |  | 34.21% | 40.54% | 0.0668 |
| Forehead (%) |  | 21.58% | 25.80% | 0.1646 |
| Pars orbitalis (%) |  | 14.74% | 12.04% | 0.2661 |
| Ear (%) |  | 4.74% | 3.69% | 0.4622 |
| Occiput (%) |  | 30.79% | 34.40% | 0.2807 |
| Neck (%) |  | 1.58% | 2.46% | 0.3831 |
| Accompany Symptom | | | | |
| Nausea* (%) |  | 68.42% | 86.98% | < 0.0001 |
| Vomit* (%) |  | 48.42% | 66.34% | < 0.0001 |
| Photophobia* (%) |  | 47.89% | 62.41% | < 0.0001 |
| Phonophobia* (%) |  | 56.58% | 68.55% | 0.0005 |
| Nasal Obstruction (%) | | 3.42% | 4.67% | 0.3760 |
| Lacrimation (%) |  | 4.74% | 3.93% | 0.5786 |
| Sweat (%) |  | 5.26% | 5.90% | 0.6990 |
| Upset (%) |  | 15.79% | 11.30% | 0.0654 |
| Trigger | | | | |
| Stress (%) |  | 25.53% | 24.32% | 0.6968 |
| Tiredness (%) |  | 17.37% | 21.62% | 0.1329 |
| Sleep Disturbance | none (%) | 83.95% | 79.36% | 0.0564 |
|  | occasionally (%) | 3.68% | 5.90% |  |
|  | less than half (%) | 1.32% | 3.93% |  |
|  | more than half (%) | 11.05% | 10.81% |  |
| Hot or Cold (%) |  | 14.21% | 13.76% | 0.8552 |
| Sun (%) |  | 1.84% | 3.69% | 0.1170 |
| Hormones (%) |  | 5.04% | 9.33% | 0.0645 |
| Specific Odor* (%) |  | 2.11% | 5.90% | 0.0071 |
| Noisy Environment (%) | | 5.79% | 5.90% | 0.9489 |
| Strong Light (%) |  | 5.53% | 7.37% | 0.2935 |
| Hypooxygen or closed space (%) | | 1.58% | 1.23% | 0.6756 |
| Physical Exercise (%) | | 2.63% | 2.70% | 0.9507 |
| Trigger positive (%) |  | 59.74% | 63.14% | 0.3262 |
| Mitigating Factor | | | | |
| Laying Down* (%) |  | 62.63% | 71.01% | 0.0125 |
| Dark Room* (%) |  | 38.95% | 49.14% | 0.0040 |
| Massage (%) |  | 20.26% | 25.80% | 0.0657 |
| Hot Compress (%) |  | 3.16% | 4.18% | 0.4483 |
| Cold Compress (%) |  | 2.37% | 3.69% | 0.2829 |
| Premonitory Symptom | | | | |
| Dizziness (%) |  | 13.16% | 12.04% | 0.6363 |
| Photophobia (%) |  | 10.00% | 8.85% | 0.5791 |
| Phonophobia (%) |  | 7.11% | 7.37% | 0.8857 |
| Yawn (%) |  | 6.32% | 5.16% | 0.4852 |
| Stiff Neck* (%) |  | 4.47% | 8.11% | 0.0367 |
| Fatigue (%) |  | 5.53% | 5.16% | 0.8191 |
| Drowsy (%) |  | 6.32% | 3.93% | 0.1280 |
| Dysesthesia (%) |  | 3.95% | 4.18% | 0.8706 |
| Concentration change (%) | | 3.42% | 3.69% | 0.8414 |
| Fidget (%) |  | 2.37% | 3.69% | 0.2829 |
| Poor Appetite* (%) |  | 5.53% | 2.70% | 0.0451 |
| Mood change (%) |  | 3.42% | 1.97% | 0.2055 |
| Irritability (%) |  | 2.37% | 1.72% | 0.5195 |
| Sensation of cold (%) | | 1.84% | 3.19% | 0.2285 |
| Due to the low occurrence rate, some variables cannot be effectively statistically compared, listed below.  Headache area: face, jaw;  Trigger: alcohol, coffee, cheese, monosodium glutamate, nitro, cough, sexual activity, menophause, position, eye tired, cervical spondylosis, starve, catch a cold;  Mitigating Factor: fast walk, stand, exercise, pregnant;  Premonitory Symptom: loquacity, thirsty, constipation, diarrhea, osmophobia, diuresis, dysphasia, craving food, over activity, edema.  Hormones induced headache included migraine induced by menstruation or pregnancy. The incidence of trigger hormones was calculated in female subjects.  Headache intensity: mild (VSA 1~3), moderate (VSA 4~6), severe (VSA 7~10).  Migraine-like Headache Type include throbbing headache, distending headache, bursting headache.  AWM : typical aura with migraine headache; AWNM : typical aura with non-migraine headache; SD: standard deviation, h: hours, d: days, m: months, y: years, d/m: days/month, y: years old.  * *p* value < 0.05. | | | | |

**Supplementary Table 5. Comparison between MWoA and AWNM**

|  |  | MWoA | AWNM | *p* value |
| --- | --- | --- | --- | --- |
| Demographic Features | | | | |
| Number |  | 4651 | 380 |  |
| Age* | Mean ± SD (y) | 39.33±11.96 | 35.46±14.65 | < 0.0001 |
|  | ≤ 18 y (%) | 3.83% | 12.63% | < 0.0001 |
|  | 19 ~ 40 y (%) | 50.66% | 52.37% |  |
|  | > 40 y (%) | 45.52% | 35.00% |  |
| Gender* | Female (%) | 80.56% | 62.63% | < 0.0001 |
| Age at Onset* | Mean ± SD (y) | 28.72±11.94 | 28.31±14.92 | 0.5350 |
|  | ≤ 18 y (%) | 22.14% | 33.16% | < 0.0001 |
|  | 19 ~ 40 y (%) | 60.14% | 46.32% |  |
|  | > 40 y (%) | 15.59% | 20.53% |  |
| Headache Features | | | | |
| Migraine-like Headache Type* (%) | | 91.87% | 83.68% | < 0.0001 |
| Headache Intensity* | VAS mean ± sd | 7.13±1.62 | 6.24±1.97 | < 0.0001 |
|  | mild (%) | 1.18% | 9.47% | < 0.0001 |
|  | moderate (%) | 32.79% | 44.74% |  |
|  | severe (%) | 66.03% | 45.79% |  |
| Frequency Per Month* | < 1 d/m (%) | 70.61% | 56.84% | < 0.0001 |
|  | 1~15 d/m (%) | 20.86% | 12.63% |  |
|  | > 15 d/m (%) | 15.76% | 35.26% |  |
| Aggravation by activity* | none (%) | 15.76% | 35.26% | < 0.0001 |
|  | occasionally (%) | 8.56% | 8.68% |  |
|  | less than half (%) | 7.33% | 6.05% |  |
|  | more than half (%) | 68.35% | 50.00% |  |
| Headache Course* | Median (m) | 106.50 | 45.00 | < 0.0001 |
| Headache Duration* | Mean ± SD (h) | 25.97±29.83 | 12.14±45.53 | < 0.0001 |
| Family History* (%) | | 46.91% | 36.05% | 0.0003 |
| Main Headache Area | | | | |
| Tempus* (%) |  | 69.28% | 58.95% | < 0.0001 |
| Top* (%) |  | 39.91% | 34.21% | 0.0290 |
| Forehead (%) |  | 23.35% | 21.58% | 0.4318 |
| Pars orbitalis (%) | | 12.17% | 14.74% | 0.1438 |
| Ear (%) |  | 4.84% | 4.74% | 0.9298 |
| Occiput (%) |  | 35.11% | 30.79% | 0.0890 |
| Neck (%) |  | 2.37% | 1.58% | 0.3262 |
| Accompany Symptom | | | | |
| Nausea* (%) |  | 87.49% | 68.4% | < 0.0001 |
| Vomit* (%) |  | 59.49% | 48.4% | < 0.0001 |
| Photophobia* (%) | | 61.97% | 47.9% | < 0.0001 |
| Phonophobia* (%) | | 70.63% | 56.6% | < 0.0001 |
| Nasal Obstruction* (%) | | 1.68% | 3.42% | 0.0142 |
| Lacrimation* (%) | | 2.56% | 4.74% | 0.0121 |
| Sweat* (%) |  | 1.16% | 5.26% | < 0.0001 |
| Upset* (%) |  | 6.49% | 15.8% | < 0.0001 |
| Trigger | | | | |
| Stress* (%) |  | 30.83% | 25.53% | 0.0307 |
| Tired* (%) |  | 24.88% | 17.37% | 0.0010 |
| Sleep Difficulty* (%) | none | 80.03% | 83.95% | 0.0499 |
|  | occasionally | 3.35% | 3.68% |  |
|  | less than half | 3.87% | 1.32% |  |
|  | more than half | 12.75% | 11.05% |  |
| Hot or Cold (%) | | 15.33% | 14.21% | 0.5594 |
| Sun (%) |  | 2.73% | 1.84% | 0.3010 |
| Hormones* (%) | | 10.14% | 5.04% | 0.0104 |
| Specific Odor* (%) | | 6.51% | 2.11% | 0.0006 |
| Noisy Environment (%) | | 5.59% | 5.79% | 0.8710 |
| Strong Light* (%) | | 3.03% | 5.53% | 0.0081 |
| Alcohol * (%) |  | 2.04% | 0.53% | 0.0388 |
| Hypooxygen or closed space (%) | | 2.19% | 1.58% | 0.4271 |
| Physical Exercise (%) | | 2.92% | 2.63% | 0.9515 |
| Mitigating Factor | | | | |
| Laying down (%) | | 66.31% | 62.63% | 0.1456 |
| Dark room (%) | | 43.13% | 38.95% | 0.1131 |
| Massage* (%) |  | 25.65% | 20.26% | 0.0201 |
| Hot Compress (%) | | 5.01% | 3.16% | 0.1068 |
| Cold Compress (%) | | 2.45% | 2.37% | 0.9201 |
| Pregnant (%) |  | 2.30% | 1.10% | 0.5361 |
| Premonitory Symptom | | | | |
| Dizziness* (%) |  | 6.51% | 13.16% | < 0.0001 |
| Photophobia* (%) | | 2.15% | 10.00% | < 0.0001 |
| Phonophobia* (%) | | 2.15% | 7.11% | < 0.0001 |
| Yawn* (%) |  | 4.04% | 6.32% | 0.0339 |
| Stiff Neck* (%) | | 8.28% | 4.47% | 0.0085 |
| Fatigue* (%) |  | 2.39% | 5.53% | 0.0002 |
| Drowsy* (%) |  | 3.55% | 6.32% | 0.0064 |
| Dysesthesia* (%) | | 1.68% | 3.95% | 0.0016 |
| Irritability (%) | | 1.40% | 2.37% | 0.1306 |
| Fidget (%) |  | 1.29% | 2.37% | 0.0822 |
| Poor Appetite* (%) | | 1.51% | 5.53% | < 0.0001 |
| Concentration change* (%) | | 1.44% | 3.42% | 0.0030 |
| Due to the low occurrence rate, some variables cannot be effectively statistically compared, listed below.  Headache Area: face, jaw;  Trigger: coffee, cheese, monosodium glutamate, nitro, cough, sexual activity, menophause, position, eye tired, cervical spondylosis, starve, catch a cold;  Mitigating Factor: stand, fast walk, exercise;  Premonitory Symptom: mood change, sensation of cold, loquacity, thirsty, constipation, diarrhea, osmophobia, diuresis, dysphasia, craving food, over activity, edema.  Hormones induced headache included migraine induced by menstruation or pregnancy. The incidence of trigger hormones was calculated in female subjects.  Headache intensity: mild (VSA 1~3), moderate (VSA 4~6), severe (VSA 7~10).  Migraine-like Headache Type include throbbing headache, distending headache, bursting headache.  MWoA : migraine without aura; AWNM : typical aura with non-migraine headache; SD: standard deviation, h: hours, d: days, m: months, y: years, d/m: days/month, y: years old.  * *p* value < 0.05. | | | | |

**Supplementary table 6.** **Multivariate analysis of clinical characteristics for distinction of MwoA and MwA.**

| Variable | B | Wald | OR with 95%CI | *P* Value |
| --- | --- | --- | --- | --- |
| Headache Intensity | -0.08 | 9.102 | 0.923(0.876~0.972) | 0.0026 |
| Headache Duration | -0.016 | 37.235 | 0.985(0.98~0.989) | < 0.001 |
| Age at Onset | -0.016 | 17.952 | 0.984(0.977~0.991) | < 0.001 |
| Female | -0.55 | 33.028 | 0.577(0.479~0.697) | < 0.001 |
| Headache Type | -0.478 | 12.322 | 0.62(0.477~0.813) | < 0.001 |
| Frequency Per Month | -0.939 | 135.047 | 0.391(0.333~0.458) | < 0.001 |
| Aggravation by Activity | -0.102 | 7.894 | 0.903(0.842~0.97) | 0.0050 |
| Headache on Tempus | -0.279 | 9.473 | 0.756(0.633~0.904) | 0.0021 |
| Family History | -0.275 | 9.591 | 0.76(0.638~0.904) | 0.0020 |
| T.- Sleep Disturbance | -0.111 | 6.141 | 0.895(0.818~0.976) | 0.0132 |
| T.- Strong Light | 0.836 | 17.223 | 2.308(1.541~3.401) | < 0.001 |
| T.- Alcohol | -1.38 | 8.995 | 0.252(0.092~0.572) | 0.0027 |
| T.- Tiredness | -0.336 | 9.052 | 0.714(0.572~0.887) | 0.0026 |
| AS.- Nausea | -0.716 | 40.672 | 0.489(0.393~0.61) | < 0.001 |
| AS.- Phonophobia | -0.35 | 13.679 | 0.704(0.585~0.849) | < 0.001 |
| AS.- Sweat | 1.346 | 28.022 | 3.843(2.324~6.311) | < 0.001 |
| AS.- Upset | 0.547 | 13.692 | 1.729(1.288~2.301) | < 0.001 |
| PS.- Dizziness | 0.512 | 11.444 | 1.668(1.234~2.234) | < 0.001 |
| PS.- Photophobia | 1.415 | 52.002 | 4.116(2.795~6.038) | < 0.001 |
| MwoA : migraine without aura; MwA : migraine with aura; OR : odds ratio; CI : confidence interval; T. : Trigger; AS. : accompany symptom; PS. : premonitory symptom. | | | | |

**Supplementary table 7.** **Multivariate analysis of clinical characteristics for distinction of AWM and AWNM.**

| Variable | B | Wald | OR with 95%CI | *P* Value |
| --- | --- | --- | --- | --- |
| Headache Intensity | -0.198 | 19.794 | 0.821(0.752~0.895) | <0.001 |
| Headache Duration | -0.013 | 10.963 | 0.987(0.98~0.994) | <0.001 |
| Age at Onset | 0.002 | 4.694 | 1.002(1~1.003) | 0.0303 |
| Aggravation by Activity | -0.311 | 24.28 | 0.733(0.647~0.829) | <0.001 |
| Specific Odor | -0.748 | 2.728 | 0.474(0.184~1.105) | 0.0986 |
| AS.- Nausea | -0.779 | 14.967 | 0.459(0.308~0.679) | <0.001 |
| AS.- Sweat | -0.705 | 3.439 | 0.494(0.232~1.036) | 0.0637 |
| AS.- Upset | 0.425 | 2.969 | 1.529(0.946~2.492) | 0.0848 |
| AWM : typical aura with migraine headache; AWNM : typical aura with non-migraine headache; OR : odds ratio; CI : confidence interval; AS. : accompany symptom. | | | | |

**Supplementary table 8. Fit statistics for the selected tree.**

|  |  | Leaves | Sensitivity | Specificity | Youden’s index | AUC | Predictors (ranked by their importance) | |
| --- | --- | --- | --- | --- | --- | --- | --- | --- |
| MWoA vs MWA | Train | 4 | 0.9967 | 0.0584 | 0.0551 | 0.6352 | Sex > Headache duration > Frequency | |
|  | Test | 4 | 0.9962 | 0.0857 | 0.0819 | 0.6002 |  |  |
| AWNM vs AWM | Train | 2 | 0.6395 | 0.8198 | 0.4593 | 0.7297 | Duration | |
|  | Test | 2 | 0.6042 | 0.7945 | 0.3987 | 0.6993 |  |  |
| MWoA vs AWNM | Train | 8 | 0.9896 | 0.3179 | 0.3075 | 0.8137 | Duration > Frequency > Sex | |
|  | Test | 8 | 0.9895 | 0.2468 | 0.2363 | 0.8147 |  |  |
| MWoA : migraine without aura; MWA : migraine with aura; AWM : typical aura with migraine headache; AWNM : typical aura with non-migraine headach. | | | | | | | |  |
